# A Rare Case of Acute Pericarditis as a Primary Presentation of Differentiation Syndrome

**DOI:** 10.7759/cureus.24213

**Published:** 2022-04-17

**Authors:** Bandar Alyami, Anas A Alharbi, Brijesh Patel

**Affiliations:** 1 Medicine, West Virginia University School of Medicine, Morgantown, USA; 2 Medicine, West Virginia University, Morgantown, USA; 3 Cardiology, West Virginia University School of Medicine, Morgantown, USA

**Keywords:** arsenic trioxide (ato), all-trans retinoic acid (atra), acute myeloid leukemia (aml), differentiation syndrome, acute pericarditis

## Abstract

A 47-year-old male with a history of acute promyelocytic leukemia was admitted for his induction chemotherapy session with all-trans retinoic acid and arsenic trioxide. On day 25, his medical course became complicated with differentiation syndrome and he developed isolated acute pericarditis.

## Introduction

Acute promyelocytic leukemia (APL) is an aggressive type of acute myeloid leukemia (AML) with a high mortality rate. However, since the introduction of differentiating agents such as all-trans retinoic acid (ATRA) and arsenic trioxide (ATO) as induction chemotherapy, the mortality rate has highly reduced with the total recovery of most patients [1]. In a clinical trial of APL patients who received ATRA, De Botton et al. found the incidence of the retinoic acid syndrome, now called differentiation syndrome (DS), was 15%. He also reported that 89% of patients with DS developed respiratory distress and only 19% had a pericardial effusion [2]. DS is a life-threatening complication if not treated promptly. Signs and symptoms are often nonspecific and may include dyspnea, pulmonary interstitial infiltrates, fever, hypotension, acute renal failure, and pleuropericardial effusion [3]. We are presenting a rare case of acute pericarditis secondary to DS.

## Case presentation

**Figure 1:**
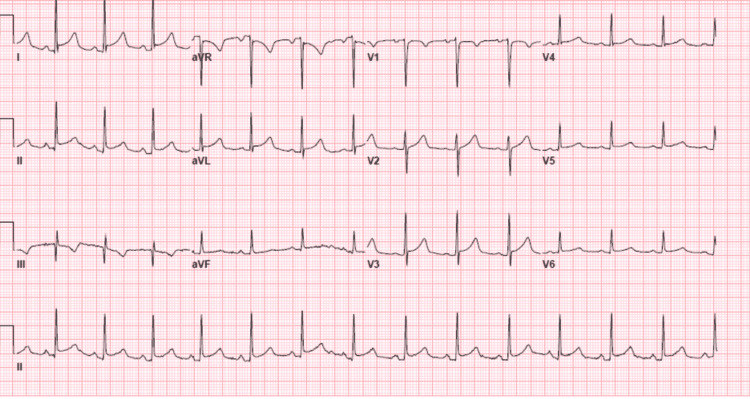
Twelve-lead electrocardiogram showing diffuse ST elevations and PR depression.

**Figure 2:**
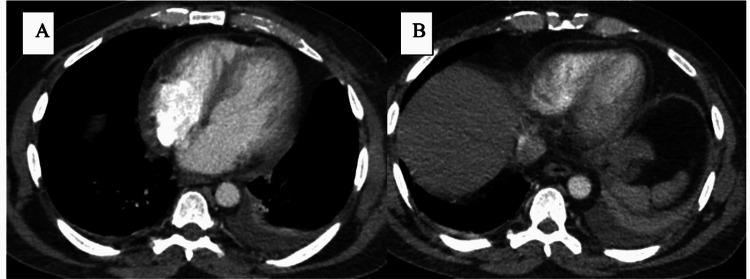
Chest CT showing mild pericardial effusion and left side pleural effusion.

**Figure 3:**
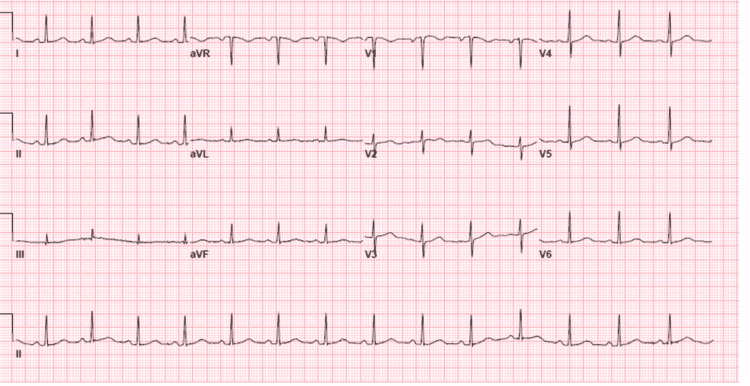
Twelve-lead electrocardiogram showing normal sinus rhythm with the resolution of ST and PR segments abnormalities.

A 47-year-old male diagnosed with APL was admitted to a cancer center. Induction therapy consisting of ATRA and ATO was initiated. The patient had been well until the 25th day from the initiation of therapy, but then he began experiencing chest pain in the substernal region that was not radiating and improved when he leaned forward. The cardiology team was consulted to evaluate the chest pain. The patient had a history of hypertension and seizure disorder. His medications were hydrochlorothiazide and an anti-seizure drug (ASD). He did not report a history of smoking or drug abuse. His family history revealed coronary artery disease (CAD) in his father. Findings on a screening electrocardiogram (ECG) were normal. Transthoracic echocardiography (TTE) before the induction therapy revealed a normal left ventricle ejection fraction (LVEF) with no pericardial effusion. His temperature was 37°C, blood pressure was 118/64 mmHg, heart rate was 88 beats/min, respiratory rate was 18 breaths/min, and oxygen saturation was 96%. The patient was looking acutely ill and there was no pericardial friction rub or rales on the chest exam. An ECG revealed sinus rhythm with diffuse ST-segment elevation and PR depression (Figure 1). TTE showed normal LVEF without wall motion abnormality or significant valvular disease, but there was a small pericardial effusion. Laboratory test findings indicated normal troponin level, pancytopenia, C-reactive protein (CRP) level of 164 mg/dl (range: 0-0.8 mg/dl), and erythrocyte sedimentation rate (ESR) of 87 mm/hr (range: 0-15 mm/hr). Computed tomography (CT) of the chest with contrast was unremarkable for pulmonary embolism (PE), but there was small left pleural effusing and mild pericardial effusion (Figure 2). In view of normal troponin and normal cardiac function on TTE, we continued the ATRA and ATO therapy with caution and continuous monitoring. A diagnosis of acute pericarditis was made based on the characteristic chest pain and ECG findings, in addition to the pericardial effusion and elevated CRP. He was diagnosed and treated for acute pericarditis. The patient experienced some relief from symptoms after being given Toradol for acute pericarditis and steroids for DS. He was also started on oral colchicine 0.6 mg once per day for three months. The patient’s symptoms subsided over the course of treatment with the resolution of the ECG’s changes during monitoring, and no further imaging was performed. The patient was completely asymptomatic, and his ECG showed resolution of ST-segment elevation and PR depression three days after the initiation of colchicine (Figure 3). He followed up with the cardio-oncology clinic as an outpatient, and he will be maintained on colchicine for three months.

## Discussion

APL DS is recognized when patients with APL are treated with ATRA and/or ATO. Frankel et al. reported the first case of DS in 1992, describing it as a potentially lethal syndrome if not diagnosed and treated promptly [3]. To date, DS has no specific criteria for the diagnosis, and it requires a high index of suspicion as the presentation can be variable. Common symptoms and findings upon presentation include dyspnea, fever, chest pain, hypotension, pleuropericardial effusion, and acute kidney injury.

In DS, reported cases have shown cardiac involvement [4-13]. Myopericarditis was described in four cases, myocarditis was present in five cases, while the involvement of entire cardiac wall layers was seen in one case. To the best of the authors’ knowledge, this is the first reported case of isolated pericarditis as a cardiac involvement with DS. Acute pericarditis is most often associated with viral infection or is idiopathic. According to the European Society of Cardiology (ESC) clinical practice guidelines, the clinical diagnosis of acute pericarditis can be made with two out of four criteria including chest pain, pericardial rub, newly widespread ST-elevation or PR depression on the ECG, and pericardial effusion [14]. CRP is an important inflammatory marker when acute pericarditis is clinically suspected, and it plays an important role in the monitoring of the disease’s progression and treatment response. Non-steroidal anti-inflammatory drugs (NSAIDs) and colchicine are considered the primary treatment, with corticosteroids considered as an alternative [15].

In our patient, a clinical diagnosis of acute pericarditis was made based on the presence of three criteria, in addition to the elevated inflammatory markers and recent initiation of ATRA chemotherapy. Meanwhile, we considered other differential diagnoses including CAD, PE, and myocarditis. However, the cardiac enzyme was not elevated, the EKG had no horizontal ST-elevation, the TTE had normal LVEF without wall motion abnormality, and the chest CT with contrast excluded PE.

## Conclusions

APL needs prompt initiation of induction chemotherapy with ATRA for effective treatment; however, ATRA can cause a fatal drug reaction that usually results in the development of DS. We describe a case of isolated pericarditis secondary to the development of DS, which is a fatal complication resulting from ATRA chemotherapy. To the best of our knowledge, this is the first reported case of isolated acute pericarditis as a presentation of DS. To date, DS has no specific criteria for the diagnosis, and it requires a high index of suspicion as the presentation can be variable.
